# The quality of intervention reporting in trials of therapeutic exercise for hip osteoarthritis: a secondary analysis of a systematic review

**DOI:** 10.1186/s13063-021-05342-1

**Published:** 2021-06-07

**Authors:** Louise C. Burgess, Thomas W. Wainwright, Khara A. James, Johan von Heideken, Maura D. Iversen

**Affiliations:** 1 Orthopaedic Research Institute, Bournemouth University, 89 Holdenhurst Road, Bournemouth, BH8 8EB UK; 2 Physiotherapy Department, University Hospitals Dorset NHS Foundation Trust, Bournemouth, BH7 7DW UK; 3 Department of Physical Therapy, Movement and Rehabilitation Sciences, Northeastern University, Boston, MA USA; 4 Department of Women’s and Children’s Health, Karolinska Institutet, Stockholm, Sweden; 5 Section of Clinical Sciences, Division of Rheumatology, Immunology & Allergy, Brigham & Women’s Hospital, Department of Medicine, Harvard Medical School, Boston, MA USA; 6 College of Health Professions, Sacred Heart University, Fairfield, CT USA

**Keywords:** Osteoarthritis, Hip, Exercise, Rehabilitation, Research design, Methods

## Abstract

**Background:**

Therapeutic exercise is recommended as a core treatment for hip osteoarthritis (HOA). Whilst it is widely accepted that exercise can improve pain and disability, optimal type and dose of exercise are yet to be agreed upon. This may, in part, be attributed to the wide variation and inadequate reporting of interventions within the literature. This study evaluates the quality of intervention reporting among trials of therapeutic exercise in HOA.

**Methods:**

Randomised controlled trials (RCTs) were sourced in a systematic review, completed in August 2020. Two raters independently used the Template for Intervention Description and Replication (TIDieR) and Consensus on Exercise Reporting Template (CERT) to evaluate intervention reporting. Correlations between quality assessment scores and CERT and TIDieR scores evaluated the relationship between internal validity and external applicability. The year of publication was compared to the quality of reporting scores.

**Results:**

Fourteen RCTs were included in the analysis. On average, studies were awarded 9.43 ± 1.95 out of 12 points for the TIDieR checklist (range 4–12) and 13.57 ± 4.01 out of 19 points for the CERT (range 5–19). Pearson’s correlation coefficient suggested that the quality of reporting had improved over time and that there was a fair, positive relationship between internal validity and external applicability.

**Discussion:**

Whilst the quality of intervention reporting is improving, many RCTs of therapeutic exercise in HOA lack the detail necessary to allow accurate evaluation and replication. Researchers are encouraged to utilise the standardised reporting guidelines to increase the translation of effective interventions into clinical practice.

**Supplementary Information:**

The online version contains supplementary material available at 10.1186/s13063-021-05342-1.

## Background

Hip osteoarthritis (HOA) is a leading cause of disability worldwide, and the prevalence continues to increase due to the world’s ageing population and the global obesity epidemic [1]. Therapeutic exercise, broadly defined as bodily movement prescribed to correct impairment, improve musculoskeletal function or maintain a state of well-being [2], is recommended as a core treatment for HOA, irrespective of age, comorbidity, pain severity or disability [3–6]. Systematic reviews of randomised controlled trials (RCTs) have found consistent evidence favouring exercise over control (no exercise or usual care) for reducing pain and improving physical function for individuals with HOA [7–9]. However, therapeutic exercise is a broad term that can encompass a large amount of variability in terms of exercise type, dose and delivery. These parameters can influence the patient’s response to exercise and the overall effectiveness of the treatment. In HOA, optimal exercise prescription can vary depending upon the individual characteristics of the patient, for example, age, weight, baseline fitness level, disease severity and comorbidities. Appropriate exercise prescription is important not only to maximise outcome improvement [10], but also to reduce the risk of symptom flare ups or exercise-related injury and increase adherence to the exercise intervention [11]. Nonetheless, there is high variability in the exercise content prescribed and evaluated in the literature, and optimal exercise dosage for patient subgroups is yet to be agreed upon [12, 13].

High variability in exercise dose for osteoarthritis may be due to structural influences (such as health systems or funding models) or provider preferences (such as differences in facility-based, regional or national preferences). Furthermore, variability within the evidence base may exist due to the differences in the study design, population, access to facilities and level of supervision. It is possible that a lack of consensus on optimal exercise dose can, in part, be attributed to inadequate reporting of interventions within research studies in arthritis [14]. Published studies of exercise interventions often lack the level of detail necessary to ascertain exercise dose and its impact on health outcomes [15]. The replicability of effective interventions is reliant upon an accurate and detailed description of the interventions’ content and delivery [16]. Many exercise interventions include an unsupervised, home-based exercise component. For patients who are new to exercise, and who are not provided with specific instructions or strategies to ensure treatment adherence and fidelity, improvement may be limited. In addition, without a complete published description of the intervention, other researchers cannot build upon findings, and clinicians may be left unclear on how to effectively implement it [17]. Hence, complete and explicit reporting of the components of the intervention is essential to ensure research findings are translated into clinical practice [18].

The Enhancing the QUAlity and Transparency Of health Research (EQUATOR) Network is an international initiative that seeks to improve the reliability and value of published health research by promoting transparent reporting through robust guidelines [19]. The Template for Intervention Description and Replication (TIDieR) [17] and the Consensus on Exercise Reporting Template (CERT) [18] checklists are promoted by the EQUATOR Network to encourage authors to report a full and accurate description of non-pharmacological interventions. Specifically designed for exercise interventions, the CERT aims to increase the clinical uptake of effective exercise programmes, enable research replication, reduce research waste and improve patient outcomes [18]. The aim of this article is to systematically evaluate the quality of intervention reporting among RCTs of therapeutic exercise in HOA using the TIDieR and CERT checklists. Items on each checklist that are not commonly reported are identified and highlighted as areas to improve future reporting.

## Methods

This is a secondary analysis of a systematic review that evaluated the reporting of adverse events in RCTs of therapeutic exercise for HOA [20]. Randomised controlled trials of therapeutic exercise for managing HOA were sourced in the systematic review, registered a priori on the International Prospective Register of Systematic Reviews (PROSPERO registration number: CRD42019136454) [21]. The review found that the exercise-related risk for harm was minimal for individuals with HOA; however, reporting of adverse events was inconsistent in the literature [20]. A full description of the protocol specifying the data sources, search strategy, eligibility criteria and study selection can be found within the review [20], reported in accordance with the Preferred Reporting Items for Systematic Reviews and Meta-Analyses (PRISMA) statement [22].

**Fig. 1:**
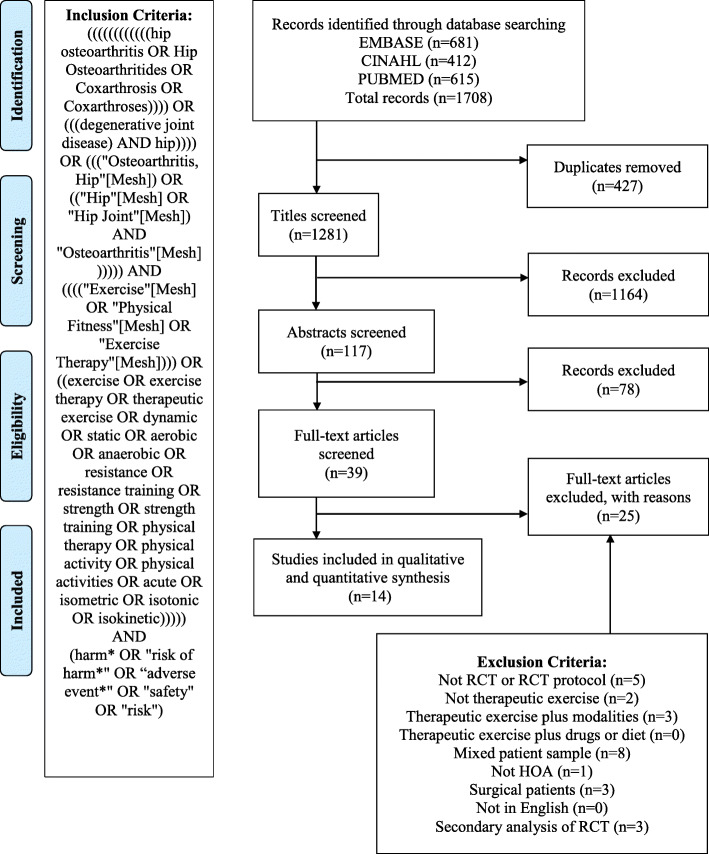
PRISMA flowchart of study selection [20]

In brief, a web-based literature search was completed in August 2020, and the electronic databases sourced included the Cochrane Library, CINAHL Complete, PubMed and EMBASE. A search strategy was developed to capture RCTs that had evaluated a trial of therapeutic exercise in adults diagnosed with HOA between 1 January 1980 and 1 August 2020. Secondary searching was also undertaken, whereby the reference lists of the yielded articles were searched for relevant citations. Studies were included if they were conducted in a cohort of adults (aged over 18 years) with osteoarthritis of the hip and met the predetermined eligibility criteria [20]. Studies that included several non-pharmacological interventions were considered eligible providing the therapeutic exercise arm included only therapeutic exercise or exercise combined with education. Studies were excluded if they were not a RCT, were not therapeutic exercise, were therapeutic exercise plus another modality other than education, were secondary analyses of a RCT, or included any participants with a history of arthroplasty and/or participants with other forms of arthritis (knee osteoarthritis, rheumatoid arthritis) and did not report separate outcomes for participants based on their diagnosis. A PRISMA flowchart describing the study selection process can be found in Fig. 1.

### Quality assessment

The Physiotherapy Evidence Database (PEDro) scale (1999) was used to critically appraise the studies included within our search. The methodological quality of the RCTs was determined independently by three researchers (KAJ, JvH and MDI), and any discrepancies were resolved through discussion. The eleven-item scale is a valid measure used to assess RCTs [23, 24] with each study scored out of ten, with a score of 6 as the threshold for a high-quality study (item 1 on the scale indicates external validity). The PEDro scale scores ten items: random allocation, concealed allocation, similarity at baseline, subject blinding, therapist blinding, assessor binding, greater than 85% follow-up for at least one key outcome, intention-to-treat analysis, between-group statistical comparison for at least one key outcome, and point and variability measures for at least one key outcome [23].

### Quality of intervention reporting

Data were collected on the quality of reporting of therapeutic exercise interventions using the TIDieR [17] and the CERT [18] checklists. Whilst the TIDieR and CERT were only published in 2014 and 2016, respectively, the criteria included in each checklist represent long-standing examples of best practice in research, and thus, they were considered suitable tools for this analysis. The TIDieR (supplementary material 1) is a 12-item checklist, developed as an extension of the CONSORT 2010 statement (item 5) [25] and the SPIRIT 2013 statement (item 11) [26]. It was created to address the deficiencies identified in the reporting of non-pharmacological interventions, which are thought to reduce the potential impact of research on clinical practice. The checklist criteria include a brief name, a rationale for delivering the intervention, a description of intervention materials and procedures, intervention provider, delivery method and setting, detail on exercise dose, whether the intervention was tailored or modified, and methods to monitor adherence or fidelity [17].

The CERT (supplementary material 2) was created as an extension of the TIDieR and provides guidance on the minimum set of key items considered essential to report replicable exercise programmes [18]. It was developed using a meta-epidemiological review of exercise interventions for chronic health conditions and thus was considered an appropriate tool to evaluate therapeutic exercise interventions for adults with HOA. The checklist includes 16 items listed under seven domains: what (materials), who (provider), how (delivery), where (location), when and how much (dosage), tailoring (what, how), and how well (compliance/planned and actual), with a maximal attainable score of 19 [18]. Several of the items described in the TIDieR and CERT checklists overlap. However, the CERT was designed so that overlapping items were aligned with the TIDieR, and thus, each checklist was analysed independently. The CERT extends the recommendations made in the TIDieR by seeking more information about the type of exercise, dose, intensity, frequency, and supervision requirements [27]. Furthermore, when an individualised treatment is prescribed, the CERT requests information on how the exercise is tailored [27].

### Data extraction

Data were extracted from the included manuscripts into extraction sheets developed in Microsoft Excel using the proforma provided in the TIDieR and CERT guidance documents [17, 18]. Data were extracted on the item details, the location of the item, item score (‘yes’ or ‘no’), and reason for rating. Only published data on the exercise description were extracted; no attempts were made to contact the authors to retrieve additional information in cases it was missing from the manuscript. Whilst it may have been possible to contact the authors, doing so would not be efficient for clinicians who were trying to replicate the exercise intervention and is thus not realistic of clinical practice. However, additional information provided in clearly cited and accessible preliminary studies, published protocols, or supplementary materials was explored for further details when the primary study lacked information. Items were scored with a ‘no’ if the item was missing from the manuscript or lacked sufficient detail to allow replication. Likewise, items were considered incomplete if they were only partially described (for example, in the TIDieR checklist, items 5, 6, and 8 were only awarded a ‘yes’ if all elements of the criteria were met). All studies were assessed by two independent reviewers (TW and LB). Where discrepancies occurred between the reviewers’ scores, discussion with the wider research team (KAJ, JvH, and MDI) was used to resolve the disagreement.

### Data analysis

Convergent validity between the TIDieR and CERT checklists was assessed using Pearson’s correlation coefficient [28]. Quality assessment scores were compared to the quality of reporting scores using Pearson’s correlation, to evaluate whether there was a relationship between internal validity and external applicability. In addition, the year of publication was compared to total TIDieR and CERT scores, to evaluate whether the quality of intervention reporting had increased in recent years. Correlation coefficients were interpreted using definitions from Chan [29]. The results were presented in a descriptive analysis, and all data were analysed using IBM SPSS Statistics version 26 (SPSS Inc., Chicago, USA).

## Results

### Study characteristics

The search yielded fourteen RCTs with a total of 707 participants enrolled in an intervention of therapeutic exercise for HOA [30–43]. These studies are described in detail in the original systematic review [20]. Briefly, the mean age of participants was 62.4 years, and 67% of the population were female. Four studies were conducted with patients with end-stage HOA and the remaining with patients with earlier stages of the disease. The median number of participants per therapeutic exercise arm was 36 (range 16–70).

### Quality of intervention reporting

**Fig. 2:**
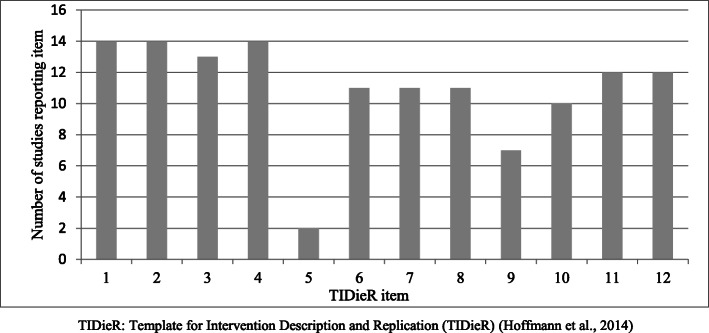
The number of studies reporting TIDieR items. TIDieR, Template for Intervention Description and Replication [17]

**Fig. 3:**
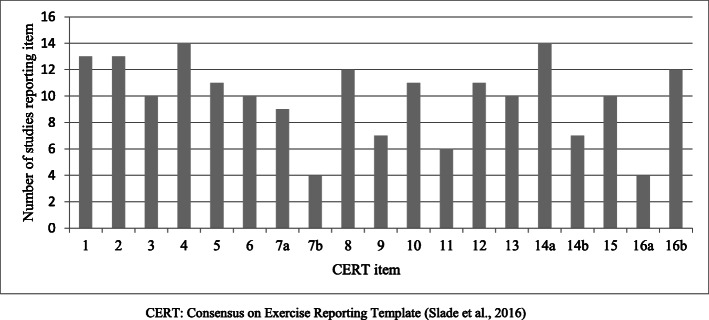
The number of studies reporting CERT items. CERT, Consensus on Exercise Reporting Template [18]

On average, studies were awarded 9.43 ± 1.95 points out of a possible 12 points for the TIDieR checklist (range 4–12) and 13.57 ± 4.01 out of a possible 19 points for the CERT (range 5–19). Convergent validity between the two assessment tools was very strong (r = 0.86, p < 0.001). One study reported all items on both the TIDieR and CERT [31], and the lowest-scoring study reported four and five items of the TIDieR and CERT checklists, respectively [36]. The number of studies reporting TIDieR and CERT items are demonstrated in Table 1 and Figs. 2 and 3.

**Table 1:** TIDieR and CERT scores of RCTs of therapeutic exercise in hip osteoarthritis

Study	TIDieR													CERT																			
	1	2	3	4	5	6	7	8	9	10	11	12	Total	1	2	3	4	5	6	7a	7b	8	9	10	11	12	13	14a	14b	15	16a	16b	Total
Bearne et al. 2011 [30]	1	1	1	1	0	1	1	1	0	1	1	1	**10**	1	1	1	1	1	1	0	0	0	0	1	0	1	0	1	0	0	0	1	**10**
Bennell et al. 2018 [31]	1	1	1	1	1	1	1	1	1	1	1	1	**12**	1	1	1	1	1	1	1	1	1	1	1	1	1	1	1	1	1	1	1	**19**
Bieler et al. 2017 [32]	1	1	1	1	0	1	1	1	1	1	1	1	**11**	1	1	1	1	1	1	1	1	1	1	1	0	1	1	1	1	1	1	1	**18**
Fernandes et al. 2010 [33]	1	1	1	1	0	1	1	1	1	1	1	1	**11**	1	1	1	1	1	1	1	1	1	0	1	1	1	1	1	1	1	0	1	**17**
French et al. 2013 [34]	1	1	1	1	1	1	1	1	0	0	1	1	**10**	1	1	1	1	1	1	1	0	1	1	1	0	1	1	1	0	1	1	1	**16**
Fukumoto et al. 2014 [35]	1	1	1	1	0	1	1	1	1	0	1	1	**10**	1	1	1	1	1	1	1	0	1	1	0	0	1	1	1	1	1	0	1	**15**
Gocen et al. 2004 [36]	1	1	0	1	0	0	0	0	1	0	0	0	**4**	0	1	0	1	0	0	0	0	1	0	0	1	0	0	1	0	0	0	0	**5**
Hermann et al. 2016 [37]	1	1	1	1	0	1	1	1	0	1	1	1	**10**	1	1	1	1	1	0	1	0	1	0	1	1	1	1	1	0	0	0	1	**13**
Hoeksma et al. 2004 [38]	1	1	1	1	1	0	1	0	0	0	1	1	**8**	1	1	0	1	0	1	0	0	0	0	1	1	1	0	1	0	1	1	1	**11**
Osteras et al. 2017 [39]	1	1	1	1	0	1	0	1	0	1	1	1	**9**	1	1	0	1	1	0	0	0	1	0	0	0	0	1	1	0	1	0	1	**9**
Rooks et al. 2006 [40]	1	1	1	1	0	0	1	1	0	1	1	1	**9**	1	1	0	1	1	1	1	1	1	0	1	1	1	1	1	0	1	0	1	**15**
Shrier et al. 2008 [41]	1	1	1	1	0	1	1	1	0	1	0	0	**8**	1	0	1	1	0	0	1	1	1	1	0	0	1	1	1	1	1	0	0	**12**
Tak et al. 2005 [42]	1	1	1	1	0	1	0	0	1	1	1	1	**9**	1	1	1	1	1	1	0	0	1	1	1	0	0	0	1	0	1	0	1	**12**
Thompson et al. 2020 [43]	1	1	1	1	0	1	1	1	1	1	1	1	**11**	1	1	1	1	1	1	1	1	1	1	1	1	1	1	1	1	1	0	1	**18**

*TIDieR* Template for Intervention Description and Replication [17], *CERT* Consensus on Exercise Reporting Template [18], *RCT* randomised controlled trial

All studies were awarded a point for TIDieR item 1: provide the name or phrase that describes the intervention and 2: describe the rationale behind the intervention. All studies were also awarded a point for CERT item 4: describe whether the exercises are supervised or unsupervised and item 14a: describe whether the exercises are generic or tailored. Consistently low-scoring items of the TIDieR checklist were item 5: description on the expertise, background and training of intervention provider; 7: description of the location where the intervention occurred; 9: description on how the intervention was titrated or adapted; and 10: description on how the intervention was modified during the course of the study. Consistently low-scoring items of the CERT were item 7b: description of how the exercise programme was progressed; item 9: detailed description of the home exercise component; item 11: reporting of adverse events; 14b: description of how the exercises were tailored to the individual; 15: the decision rule for determining the starting level; and 16a: description of how adherence or fidelity is assessed.

### Year of publication

**Fig. 4:**
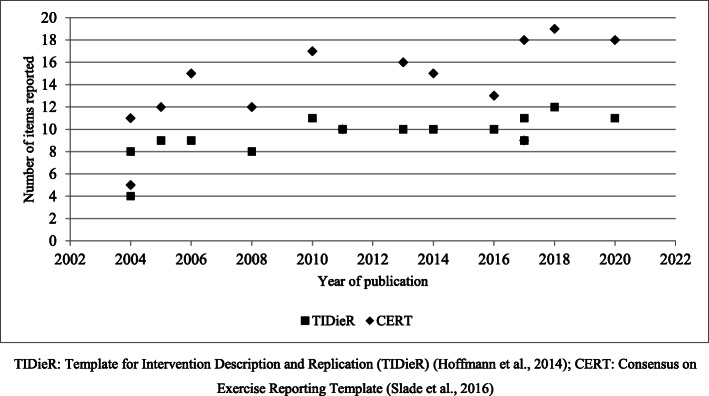
Year of publication and total CERT/TIDieR scores. TIDieR, Template for Intervention Description and Replication [17]; CERT, Consensus on Exercise Reporting Template [18]

Pearson’s correlation coefficient demonstrated a statistically significant moderate, positive relationship (r = 0.71; p = 0.004) between total TIDieR score and year of article publication. Similarly, there was a statistically significant moderate, positive relationship between total CERT score and year of publication (r = 0.57; p = 0.03), suggesting that the quality of intervention reporting has improved over time (Fig. 4).

### Quality assessment

The mean PEDro score of the included RCTs was 7.4 (range 6–10), corresponding to a high level of internal validity [44]. Pearson’s correlation coefficient demonstrated a fair, positive relationship (0.47) between total TIDieR score and total PEDro score, although was not considered statistically significant (p = 0.09). Likewise, there was a fair, positive relationship between total CERT and total PEDro score (0.49) that did not reach statistical significance (p = 0.07).

## Discussion

The evidence supporting therapeutic exercise as an efficacious treatment for HOA is ever-expanding. Nonetheless, clinical practice remains varied [45], and the most recent Cochrane review of exercise for osteoarthritis of the hip concluded that additional research is required to provide evidence of optimal exercise content and dosage [12]. To build and expand upon existing evidence, a complete published description of the intervention is required [17]. Non-pharmacological interventions in RCTs are often poorly reported [46], and our analysis suggests this remains the case among trials of therapeutic exercise in HOA. Our findings assimilate with previous investigations into the reporting of exercise interventions in studies of knee osteoarthritis and patellofemoral pain [47–49], and within non-musculoskeletal populations [50, 51]. Therapeutic exercise is a broad term and includes a number of programming variables that can be manipulated. Hence, poor reporting may be due to the complex nature and variability in prescribed treatments. However, inconsistencies in reporting and a lack of transparency around specific exercise prescription may lead to clinical uncertainty and thus impede the implementation of therapeutic exercise into practise. Moreover, future research endeavours will likely be limited by the inadequate reporting of existing evidence. Whilst the quality of reporting has improved in recent years, likely due to increased uptake of guidance provided by the EQUATOR Network, and journals requiring checklist inclusion as a prerequisite for publication, this analysis highlights some key areas for improvement in the reporting of therapeutic exercise interventions in HOA trials.

For example, just four studies were awarded a point for item 16a of the CERT checklist: description of how adherence or fidelity is assessed. Fidelity refers to the extent to which the exercise intervention occurred as the investigators intended it, as for various reasons, part or all of the exercise intervention may not be delivered as intended [18]. Our findings are consistent with previous investigations into the quality of exercise intervention reporting in knee osteoarthritis and patellofemoral pain [47–49]. Frequently, studies reported adherence in terms of programme attendance but did not mention intervention fidelity. Details were also lacking on how the interventions were modified during the course of the study (TIDieR item 10) and the providers’ expertise, background and specific training (TIDieR item 5). This information is important so the reader can understand the extent to which the intervention occurred as the investigators intended it, and whether the expertise of the provider or other characteristics affected outcomes, in order to evaluate treatment fidelity [17, 18]. Treatment fidelity has significant implications for the internal, external and construct validity and the statistical power of treatment outcome research [52]. For example, high treatment fidelity is necessary to ensure that the results of the trial can be directly attributable to the intervention and to allow a fair comparison of treatments [52]. Moreover, treatment fidelity increases the reproducibility and clinical implementation of the intervention by enhancing its external validity. Perhaps most importantly, fidelity can affect the outcome of the study itself. When building a scientific basis for clinical practice, we must be certain that a treatment has been consistently administered in order to be certain that the conclusions of the study are valid [53].

Often, clinical trials recruit homogenous populations to whom a standard intervention is delivered with little detail on how it is tailored or adapted [54]. This method of exercise prescription is not generalisable to clinical practice, and thus, it is important for research studies to provide clear and explicit detail on how the intervention was tailored to the individual. In the osteoarthritis population, pain, swelling, limited range of motion, muscle weakness, postural or gait instability and level of cardiovascular fitness are physical impairments that may affect the patient’s actual or perceived ability to participate in exercise [55]. Pain, in particular, can be a major barrier to beginning and maintaining an exercise programme [56]. Hence, when prescribing exercise in osteoarthritis, guidelines from the American College of Sports Medicine (ACSM) recommend that individual pain, stability and functional limitations should be taken into account to reduce the risk of symptom flare-ups or exercise-related injury [54]. Nonetheless, the studies included within this analysis scored poorly for CERT items 14b: detailed description of how the exercises were tailored to the individual and 15: description of the decision rule for determining the starting level of the exercise programme. Similarly, detail was often lacking on how the intervention was titrated or adapted, meaning that only seven of the studies were awarded a point for TIDieR item 9, ‘tailoring’. These details are vital when reporting interventions in RCTs to facilitate the development of individualised, patient-centred therapeutic exercise prescription in HOA. Often, studies included in this review stated that their intervention was tailored but did not describe in detail how tailoring was achieved. For example, in the study by Bearne et al. [30], the authors state: ‘The physiotherapist prescribed exercises for each participant according to their abilities, and monitored and revised the performance of these exercises.’ Whilst this statement confirms that the intervention was individualised, it does not provide sufficient details to allow replication or adaption of the programme into clinical practice.

Four of the fourteen studies were awarded a point for CERT item 7b: detailed description of how the exercise programme was progressed. Many studies stated that progression was adjusted by the intervention provider. Whilst this demonstrates that the intervention was tailored to the individual, it does not provide sufficient details to allow replication. For example, in the study by Tak et al. [42], the authors included the following statement: ‘All fitness equipment could be used at 2 levels (light and moderate) and was adjusted as the program (and participant) progressed.’ From this statement, the reader is unable to determine the decision rule for progressing exercise, or the amount of progressive overload prescribed.

Progressive overload is the gradual increase of stress placed on the body during exercise training and is necessary for long-term improvement [57]. It can be performed in several ways, including increasing duration, frequency, intensity or volume of exercise or reducing rest periods [56]. Guidelines from ACSM recommend that progression of exercise in osteoarthritis should be based upon the individual’s pain and symptoms and implemented through the increased duration of activity rather than intensity [54]. If overload occurs too slowly, it is likely that improvement will be limited and may lead to a loss of motivation for the participant. If overload occurs too quickly, the participant may be at risk of symptom flare-up or exercise-related injury. Patient beliefs about chronic pain often shape their attitudes and behaviours when managing their symptoms [58]. Hence, those who are unsure on what exercise they should participate in, and how to progress exercise without causing injury, will likely avoid activity due to fear of causing harm [58]. Thus, it is important that research studies clearly describe the decision rule for progressing exercise (CERT item 7a), in addition to a detailed description on how optimal progressive overload is achieved (CERT item 7b). Finally, less than half of the studies included a clear statement of adverse events (CERT item 11) [20]. Without sufficient reporting of adverse events, the relationship between exercise dose and harms-risk cannot be determined [20].

This analysis highlights the key areas for improvement in the reporting of therapeutic exercise interventions in HOA trials. Nonetheless, our analysis is limited by the small number and variability of RCTs investigating therapeutic exercise in HOA. Whilst this may have influenced the findings of the correlation analyses, fourteen studies are sufficient to produce meaningful results [59]. It should be acknowledged that the TIDieR and CERT checklists were published in 2014 and 2016, respectively [17, 18]. The reporting of interventions has improved in recent years, and this is likely due to the increasing awareness and uptake of reporting guidelines promoted by the EQUATOR Network. Furthermore, journals are increasingly requiring adherence to the EQUATOR checklists as a prerequisite for publication. Eight of the studies included in this review were conducted between 2004 and 2014, and therefore, the authors would not have had access to these guidelines which were published in 2014 and 2016. Despite this, the criteria included in each checklist represent long-standing examples of best practice in clinical research.

## Conclusions

Whilst the quality of intervention reporting has improved in recent years, many RCTs of therapeutic exercise in HOA lack the detail necessary to allow accurate evaluation and replication. Researchers are encouraged to utilise standardised reporting guidelines, such as the CERT and TIDieR, to increase the translation of effective exercise interventions into clinical practice. Furthermore, improved reporting of interventions will allow researchers to build upon findings and work towards developing guidelines for optimal exercise prescription within HOA.

## Supplementary Information

**Additional file 1.** The Template for Intervention Description and Replication Checklist (TIDieR) (Hoffman et al. 2014 [17]).

**Additional file 2.** The Consensus on Exercise Reporting Template (CERT) (Slade et al. 2016 [18]).

## Data Availability

Not applicable.

## Abbreviations

ACSM: American College of Sports Medicine
CERT: Consensus on Exercise Reporting Template
EQUATOR: Enhancing the QUAlity and Transparency of Health Research
HOA: Hip osteoarthritis
PEDro: Physiotherapy Evidence Database
RCT: Randomised controlled trial
TIDieR: Template for Intervention Description and Replication
