# Supplementary material for: The quality of intervention reporting in trials of therapeutic exercise for hip osteoarthritis: a secondary analysis of a systematic review
Source: Trials. 2021 Jun 7;22:388. doi: 10.1186/s13063-021-05342-1 (PMC8186100; doi:10.1186/s13063-021-05342-1)
Supplement: Supplementary file 2 — Additional file 2. The Consensus on Exercise Reporting Template (CERT) (Slade et al. 2016 [18]). [file 13063_2021_5342_MOESM2_ESM.pdf]

CERT ✓ Consensus on Exercise Reporting Template

A Checklist for what to include when reporting exercise programs

Section/Topic	Item #	Checklist item	Location **	
			Primary paper (page, table, appendix)	† Other (paper or protocol, website (URL)
**WHAT: materials**	1	Detailed description of the type of exercise equipment (e.g. weights, exercise equipment such as machines, treadmill, bicycle ergometer etc)	______	______
**WHO: provider**	2	Detailed description of the qualifications, teaching/supervising expertise, and/or training undertaken by the exercise instructor	______	______
**HOW: delivery**	3	Describe whether exercises are performed individually or in a group	______	______
	4	Describe whether exercises are supervised or unsupervised and how they are delivered	______	______
	5	Detailed description of how adherence to exercise is measured and reported	______	______
	6	Detailed description of motivation strategies	______	______
	7a	Detailed description of the decision rule(s) for determining exercise progression	______	______
	7b	Detailed description of how the exercise program was progressed		
	8	Detailed description of each exercise to enable replication (e.g. photographs, illustrations , video etc)	______	______
	9	Detailed description of any home program component (e.g. other exercises, stretching etc)	______	______
	10	Describe whether there are any non-exercise components (e.g. education, cognitive behavioural therapy, massage etc)	______	______
	11	Describe the type and number of adverse events that occurred during exercise	______	______

**WHERE: location**	12	Describe the setting in which the exercises are performed		
**WHEN, HOW MUCH: dosage**	13	Detailed description of the exercise intervention including, but not limited to, number of exercise repetitions/sets/sessions, session duration, intervention/program duration etc		
**TAILORING: what, how**	14a	Describe whether the exercises are generic (one size fits all) or tailored whether tailored to the individual		
	14b	Detailed description of how exercises are tailored to the individual		
	15	Describe the decision rule for determining the starting level at which people commence an exercise program (such as beginner, intermediate, advanced etc)		
**HOW WELL: planned, actual**	16a	Describe how adherence or fidelity to the exercise intervention is assessed/measured		
	16b	Describe the extent to which the intervention was delivered as planned		

***It is recommended that this checklist is used in conjunction with the Explanation and Elaboration Statement which is a guide each item in the CERT Checklist**

The CERT Checklist is designed for reporting details of an exercise intervention. The CERT Checklist should be used in conjunction with a reporting checklist appropriate for the study type e.g. the CONSORT Statement (www.consort-statement.org) for randomised controlled trials, the SPIRIT Statement (www.spirit-statement.org) for a clinical trial protocol. For further guidance regarding reporting guidelines please consult the EQUATOR network (www.equator-network.org)

****** Authors – please use N/A if an item is not applicable   Reviewers – please use ”?” if information is not provided or not/insufficiently reported

† If the information is not provided in the primary paper that is under consideration, please provide details of where this information is available e.g. in a published protocol, published papers (provide citation details) or on a website (provide the URL).
